# An animal model of NLRC4-associated autoinflammation and infantile enterocolitis reveals novel therapeutic strategies

**DOI:** 10.1038/s41423-025-01355-x

**Published:** 2025-10-20

**Authors:** Yuhang Wang, Joyce Z. Gao, Prajwal Gurung, Sarah P. Short, Yiqin Xiong, Scott W. Canna, Zizhen Kang

**Affiliations:** 1 Department of Pathology, University of Iowa, Iowa City, IA USA; 2 Department of Internal Medicine, University of Iowa, Iowa City, IA USA; 3 Rheumatology and Immune Dysregulation, The Children’s Hospital of Philadelphia and University of Pennsylvania Perelman School of Medicine, Philadelphia, PA USA; 4 Present Address: Pathology and Laboratory Medicine, Boston University, Boston, MA USA

**Keywords:** NLRC4 mutation, autoinflammation, infantile enterocolitis, monogenic inflammatory bowel disease, Immunological disorders, Inflammasome

## Abstract

Inflammasomes, particularly NLRC4, play crucial roles in immune responses to intracellular bacterial infections. However, gain-of-function mutations in NLRC4 are linked to severe autoinflammatory diseases, including autoinflammation with infantile enterocolitis (AIFEC). AIFEC patients who survive infancy typically have no further intestinal symptoms but retain susceptibility to macrophage activation syndrome (MAS). However, existing mouse models do not adequately replicate the inflammation observed in AIFEC patients. To better understand this, we developed a mouse model capable of conditional expression of the activating V341A mutation in NLRC4 (NLRC4-V341A KI). Global conversion to NLRC4-V341A at the germline resulted in symptoms closely mirroring those of human AIFEC, including severe infantile enterocolitis characterized by heightened intestinal inflammation, disrupted gut epithelium, compromised intestinal barrier integrity, severe diarrhea, and mortality within 10 days post-natally. Additionally, they displayed systemic autoinflammation marked by elevated levels of IL-1β, IL-18, and IL-6, alongside cytopenia and hemophagocytosis. In contrast, conditional conversion to NLRC4-V341A in adulthood caused systemic autoinflammation with only mild enterocolitis, mirroring AIFEC patients. Using this model, we demonstrated that IL-18 and TNF blockade effectively ameliorated AIFEC disease symptoms. Unexpectedly, glucose supplementation has emerged as a promising therapeutic strategy. These findings advance our understanding of AIFEC and illuminate the ways in which inflammasome activation contributes to very early onset inflammatory bowel disease (VEO-IBD) in the developing gut.

## Introduction

Inflammasomes are cytosolic multiprotein complexes that serve as central hubs of innate immune signaling. These supramolecular platforms assemble in response to perturbations in cellular homeostasis caused by microbial invasion, sterile injury, or metabolic stress. Inflammasome activation is initiated by cytosolic pattern recognition receptors (PRRs) that sense pathogen-associated molecular patterns (PAMPs), damage-associated molecular patterns (DAMPs), or homeostasis-altering molecular processes [1]. In some contexts, activation is driven by effector-triggered immunity, wherein host sensors detect pathogen-encoded activities rather than microbial motifs per se. Canonical inflammasomes typically consist of a sensor PRR—most commonly from the NLR (nucleotide-binding domain leucine-rich repeat–containing) or ALR (AIM2-like receptor) families—the adaptor protein ASC (apoptosis-associated speck-like protein containing a CARD) and caspase-1 [2]. Upon activation, the inflammasome facilitates caspase-1 autoproteolysis, resulting in the cleavage of pro–IL-1β and pro–IL-18 into their mature cytokine forms and of gasdermin D (GSDMD), whose N-terminal fragment forms membrane pores that drive pyroptosis—a highly inflammatory form of programmed cell death [2].

Numerous inflammasome sensors, including NLRP3, AIM2, NLRP1, pyrin, and NLRC4, have been identified. These sensors differ in their structural domains, ligand specificities, and downstream signaling profiles. Collectively, they constitute the “inflammasome family,” a diverse group of innate immune sensors that mediate overlapping yet nonredundant responses to a broad array of threats [2, 3]. The NAIP–NLRC4 inflammasome represents a specialized member of this family that primarily senses intracellular bacterial ligands [4]. In mice, NAIP proteins serve as direct sensors: NAIP5 and NAIP6 recognize cytosolic bacterial flagellin, NAIP2 detects the inner rod protein, and NAIP1 binds the needle protein of the bacterial type III secretion system (T3SS), which is a conserved virulence apparatus found in gram-negative pathogens such as Salmonella typhimurium, Shigella, and Pseudomonas. Upon ligand engagement, NAIPs oligomerize with NLRC4 to form an inflammasome complex that activates caspase-1 [5]. Human NAIP is more promising and capable of detecting multiple T3SS components and flagellin. Unlike some other inflammasomes, the NAIP–NLRC4 complex can function independently of ASC, although ASC enhances caspase-1 processing and cytokine release [6]. In addition to classical bacterial PAMPs, recent findings suggest that NLRC4 may also respond to sterile triggers such as lysophosphatidylcholine (LPC), hyperosmotic stress, and retroelement-derived RNAs via interaction with DDX17 [7], indicating broader roles in immune surveillance.

NLRC4 is constitutively expressed in various immune and barrier cell types, with particularly high expression in myeloid cells and intestinal epithelial cells (IECs) [8, 9]. Within the gut epithelium, NLRC4 mediates protective responses to invasive enteric bacteria by promoting infected IEC expulsion and IL-18–dependent mucosal defense. However, dysregulated or constitutively active NLRC4 signaling can be pathogenic, leading to a spectrum of autoinflammatory disorders now classified as NLRC4 inflammasomopathies [8]. In 2014, two distinct gain-of-function mutations—NLRC4 T337S and V341A—were independently identified in patients with severe early-onset inflammatory disease [10, 11]. These variants were subsequently linked to a syndrome termed autoinflammation with infantile enterocolitis (AIFEC). Since then, 14 pathogenic NLRC4 variants have been described, with clinical phenotypes ranging from AIFEC to familial cold autoinflammatory syndrome type 4 (FCAS4) and neonatal-onset multisystem inflammatory disease (NOMID). Among these, AIFEC is the most severe and frequently lethal [12–16].

AIFEC typically presents in infancy with systemic inflammation, macrophage activation syndrome (MAS), pancytopenia, and markedly elevated circulating IL-18 [10, 11]. A defining feature is the onset of infantile enterocolitis, which is characterized by chronic diarrhea, abdominal pain, villus blunting, and transmural intestinal infiltration [11]. All reported cases of NLRC4-mediated colitis have occurred in infancy [10, 11, 17, 18]; among survivors, enterocolitis tends to resolve or attenuate with age, whereas systemic autoinflammation often persists into adolescence or adulthood [16]. Despite its severity, there are no standardized therapies for AIFEC [15, 16]. Multiple gain-of-function NLRC4 mutations have been implicated in disease, but the V341A variant is particularly prevalent and mechanistically informative. Located in the HD-1 (helical domain 1) subdomain of the nucleotide-binding domain (NOD), this mutation critically alters a hydrophobic residue to maintain the autoinhibited state of NLRC4. Structural analyses suggest that the V341A substitution disrupts a “lid” that normally occludes the ADP-binding pocket, thereby facilitating constitutive ADP–ATP exchange and spontaneous inflammasome activation [11].

To elucidate AIFEC pathogenesis and facilitate therapeutic development, appropriate animal models are urgently needed. Mice harboring the NLRC4 T337S mutation exhibit elevated serum IL-18 and canonical inflammasome activation (via Pycard, Casp1, and Gsdmd) in IECs but fail to develop spontaneous colitis or systemic inflammation, even upon immune challenge [19, 20]. Thus, current models fall short in replicating the chronic intestinal and systemic pathology observed in AIFEC patients with NLRC4 V341A mutations.

In this study, we generated a conditional knock-in (KI) mouse model carrying a loxP-flanked V341A allele (GTG to GCG) in the endogenous Nlrc4 locus. Global expression of the NLRC4 V341A allele recapitulated hallmark features of AIFEC, including early-onset enterocolitis and systemic autoinflammation. When expression is temporally induced in adulthood, mice develop persistent autoinflammatory symptoms accompanied by milder colitis. Using this model, we demonstrated that pharmacologic blockade of IL-18 and TNF effectively ameliorated disease phenotypes, and notably, glucose supplementation emerged as an unexpected yet promising therapeutic strategy. Together, these findings introduce a physiologically relevant model of NLRC4 inflammasomeopathy that provides mechanistic insight into disease pathogenesis and offers a robust platform for preclinical therapeutic testing.

## Results

### NLRC4 V341A KI mice exhibit inflammasome hyperactivation

**Fig. 1:**
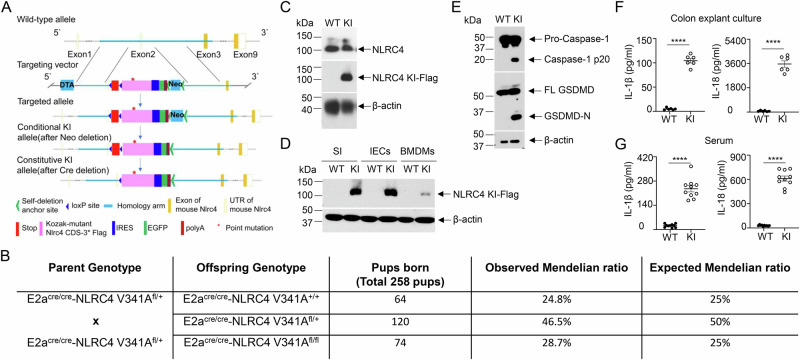
NLRC4 V341A KI mice exhibit inflammasome hyperactivation. **A** Diagram illustrating the design and generation of NLRC4 V341A-Flag KI mice. **B** Mendelian inheritance analysis of NLRC4 KI offspring as indicated. **C**–**G** Six-day-old E2aCre-NLRC4 V341A ^fl/fl^ (KI) and E2aCre-NLRC4^+/+^ (wild-type, WT) mice were used. **C** Western blot for detecting mouse NLRC4 and NLRC4-V341A-Flag expression in colon tissues of NLRC4 WT and KI mice to confirm the genotyping. **D** Western blot analysis of NLRC4 V341A-Flag expression in the indicated tissues probed with Flag and β-actin antibodies. SI small intestine; IECs intestinal epithelial cells; BMDMs bone marrow-derived macrophages. **E** Inflammasome activation in IECs from NLRC4 WT and KI mice was analyzed via western blotting with the indicated antibodies (FL GSDMD, full-length Gasdermin-D; GSDMD-N,N-terminal GSDMD. **F** IL-1β and IL-18 levels in colon explant cultures from NLRC4 WT and KI mice were measured via ELISA (*n* = 6 per group). Colon tissues were cultured in 1 ml of medium overnight, and the supernatant was collected for analysis. **G** IL-1β and IL-18 levels in NLRC4 WT and KI mouse serum were measured via ELISA (*n* = 10 per group). In (**F**, **G**), the data are shown as the mean ± SEM; ****, *p* < 0.0001 was determined by Student’s *t* test. The data are representative of at least three independent experiments

To generate NLRC4 V341A knock-in (KI) mice, we replaced the coding sequence (CDS) of exon 2 with a cassette that enables Cre-dependent expression of a Kozak-mutant Nlrc4 CDS-3*Flag-IRES-EGFP-polyA” in the target vector, introducing the V341A mutation (GTG to GCG) into the mutant Nlrc4 CDS (Fig. 1A). To achieve global expression of the KI alleles, we utilized E2a-Cre transgenic mice, which direct Cre expression to early mouse embryos [21]. E2a^Cre/Cre^ NLRC4 KI^fl/+^ mice were then bred with each other to obtain E2a-Cre NLRC4 KI^fl/fl^ (referred to as NLRC4^KI/KI^ or KI mice) and E2a-Cre NLRC4^+/+^ (wild-type control) littermate controls. The birth of NLRC4-KI mice followed Mendelian inheritance patterns (Fig. 1B).

NLRC4-KI and wild-type littermate control mice were genotyped by immunoblotting via antibodies against NLRC4 and the Flag tag. Notably, the expression of NLRC4 KI was comparable to that of endogenous NLRC4 in the colon tissue of wild-type controls (Fig. 1C). To further validate NLRC4 V341A KI expression, we analyzed KI-Flag expression in small intestine tissues, intestinal epithelial cells (IECs), and bone marrow-derived macrophages (BMDMs). We observed NLRC4 V341A KI expression across all tested tissues from KI mice but not in controls (Fig. 1D).

Given that hyperactivation of the NLRC4 inflammasome has been reported in AIFEC patients [10, 11], we investigated NLRC4 inflammasome activation in KI mice. IECs from KI mice exhibited significant caspase-1 and GSDMD cleavage, which was absent in wild-type controls (Fig. 1E), indicating hyperactivation of the inflammasome in NLRC4 KI mice. Moreover, ELISA analysis revealed significantly elevated levels of IL-1β and IL-18 in both serum and colon explant cultures from KI mice compared with those from wild-type littermate controls (Fig. 1F-G). Collectively, these data suggest that NLRC4 KI mice exhibit spontaneous hyperactivation of the NLRC4 inflammasome.

### NLRC4 V341A KI mice develop autoinflammation

**Fig. 2:**
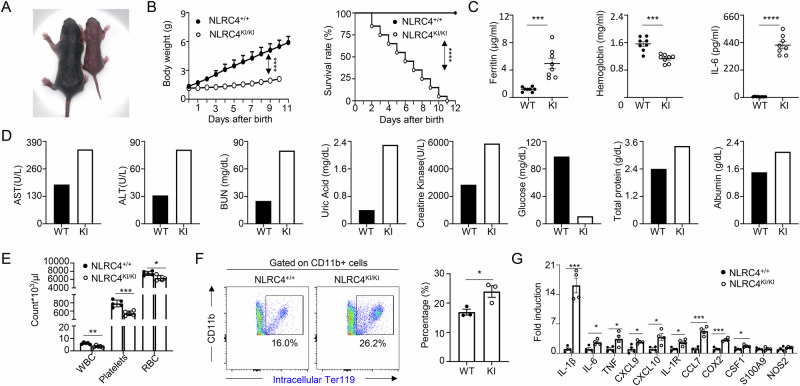
NLRC4 V341A KI mice develop autoinflammation. **A** Gross phenotype of NLRC4 WT (left) and NLRC4 KI (right) mice on day 6 after birth. **B** Body weight (left) and survival rate (right) of NLRC4 WT (NLRC4^+/+^) and NLRC4 KI (NLRC4^KI/KI^) mice after birth were plotted (*n* = 20 per group). *P* values were determined via two-way ANOVA and the Mantel‒Cox test. **C** Serum ferritin, hemoglobin and IL-6 levels in 6-day-old NLRC4 WT and NLRC4-KI mice were measured via ELISA (*n* = 8 per group). **D** Chemical parameters of blood from NLRC4 WT and KI mice were measured as indicated. ALT, alanine aminotransferase; BUN, blood urea nitrogen; LDH, lactate dehydrogenase. Sera were pooled from 8–10 6-day-old mice per group. **E** Blood cells from 6-day-old NLRC4 WT and NLRC4-KI mice were counted for the indicated cellular parameters (*n* = 5 per group). WBC, white blood cell; RBC, red blood cell. **F** Hemophagocytosis in the spleen was analyzed by flow cytometry. Splenocytes from 6-day-old NLRC4 WT and NLRC4 KI mice were first stained with fluorescence-conjugated CD11b (myeloid cells); then, the stained cells were incubated with unconjugated Ter119 antibodies to block corresponding surface antigens; and finally, the cells were permeabilized for intracellular staining of phagocytosed Ter119+ cells (*n* = 3 per group). **G** Isolated macrophages from the spleens of 6-day-old NLRC4 WT and KI pups were analyzed for inflammatory gene expression as indicated by Q-PCR (*n* = 4 per group). The data are shown as the means ± SEMs. *p* values were determined by Student’s *t* test (**C**, **E**-**G**), *p < 0.05, **p < 0.01, ***p < 0.001 and ****p < 0.0001. The data are representative of three independent experiments

Autoinflammation refers to a group of disorders characterized by uncontrolled inflammation that arises from dysregulation of the innate immune system rather than from infections, autoimmune processes, or other external triggers [22, 23]. The autoinflammation observed in AIFEC is characteristic of macrophage activation syndrome (MAS), which often manifests as cytopenia, macrophage hyperactivation, and hemophagocytosis [10, 11, 16]. We characterized the autoinflammatory phenotype in NLRC4 knock-in (KI) mice and noted profoundly decreased growth (Fig. 2A, B), with most succumbing within 10 days post-natally (Fig. 2B). Serum ELISA revealed significantly elevated levels of ferritin and IL-6 and decreased hemoglobin levels in KI mice (Fig. 2C), in addition to increased IL-1β and IL-18 levels (Fig. 1G), indicating systemic autoinflammation. Further blood biochemical analysis revealed multiorgan damage in 6-day-old KI pups, as evidenced by elevated AST and ALT levels reflecting liver damage, increased BUN and uric acid levels suggesting kidney impairment, and elevated creatine kinase levels indicative of heart damage or muscle injury (Fig. 2D). In contrast, blood glucose levels were markedly reduced in NLRC4 KI mice, reflecting hypoglycemia. However, total protein and albumin levels in the blood of KI mice were greater than those in the blood of wild-type (WT) mice, suggesting that hypoglycemia in KI mice was unlikely to be due to undernourishment (Fig. 2D).

Consistent with MAS, we detected decreased leukocyte, red blood cell, and platelet counts in the blood of NLRC4-KI mice (Fig. 2E). Intracellular staining of splenocytes followed by flow cytometric analysis revealed evidence of hemophagocytosis, as indicated by a significant increase in the frequency of Ter119+ cells among CD11b+ myeloid cells in the KI mice compared with the WT controls (Fig. 2F). Additionally, Q-PCR analysis of sorted macrophages from the spleens of NLRC4-KI and WT mice revealed that macrophages from KI mice were hyperactivated and produced significantly increased levels of proinflammatory cytokines and chemokines (Fig. 2G). Collectively, these findings suggest that NLRC4-KI mice develop autoinflammation.

### NLRC4 V341A KI mice develop infantile enterocolitis

**Fig. 3:**
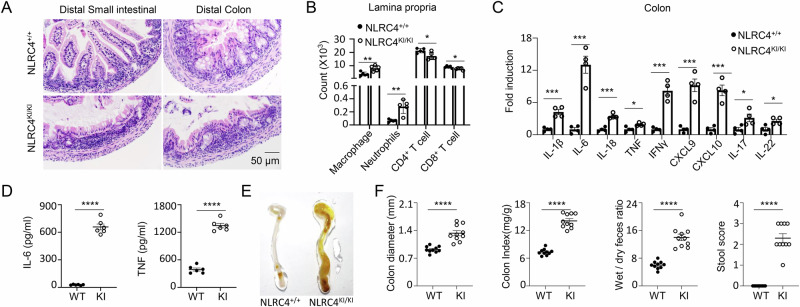
NLRC4 V341A KI mice develop infantile enterocolitis. **A** Representative H&E staining of small intestine and colon tissues from 6-day-old NLRC4 WT and KI mice as indicated. **B** Inflammatory cell infiltration in lamina propria from colon tissues of 6-day-old NLRC4 WT and KI mice was analyzed by flow cytometry (*n* = 5 per group). **C** Colon tissues from 6-day-old NLRC4 WT and KI mice were analyzed for inflammatory gene expression as indicated by real-time PCR (*n* = 4 per group). **D** IL-6 and TNF-α levels in the supernatants of colon explant cultures from 6-day-old NLRC4 WT and -KI mice were measured via ELISA (*n* = 6 per group). **E** Representative images of gross colons from 6-day-old NLRC4 WT and KI mice. **F** Colon diameter, colon index (colon weight/body weight), wet/dry feces ratio and stool score of 6-day-old NLRC4 WT and KI mice are shown as indicated (*n* = 10 per group). The data are shown as the means ± SEMs. *p* values were determined by Student’s *t* test, *p < 0.05, **p < 0.01, ***p < 0.001 and ****p < 0.0001. The data are representative of three independent experiments

A hallmark of AIFEC is infantile enterocolitis. Patients with AIFEC often present with diarrhea, abdominal pain, villus blunting, and mixed inflammatory infiltrates in the intestine [11]. To determine whether NLRC4-KI pups develop infantile enterocolitis, we collected intestinal tissues from 6-day-old NLRC4-KI and wild-type (WT) control mice. We focused our analysis on the ileum and colon since our preliminary qPCR data indicated that inflammatory gene expression was more robust in the ileum and colon of KI mice than in other intestinal segments, such as the duodenum or jejunum (data not shown). H&E staining of the distal small intestine and colon revealed villous blunting and epithelial damage, along with increased inflammatory cell infiltration in the lamina propria of KI mice (Fig. 3A). Flow cytometric analysis of mononuclear cells isolated from the colon lamina propria revealed an increase in macrophages and neutrophils, accompanied by a decrease in T cells, in the KI mice (Fig. 3B). Furthermore, compared with those from WT control mice, Q-PCR analysis of colon tissue from KI mice revealed elevated expression of proinflammatory cytokines and chemokines (Fig. 3C). ELISAs of supernatants from colon explant cultures revealed significantly increased levels of IL-6 and TNF-α in KI mice (Fig. 3D), in addition to increased levels of IL-1β and IL-18 (Fig. 1F). Notably, NLRC4-KI mice presented enlarged colons and increased colon weight (Fig. 3E, F, left two panels). These mice also presented significant diarrhea, as evidenced by a higher wet/dry feces ratio and stool score, with a higher score indicating more severe diarrhea (Fig. 3F, right two panels). Taken together, these findings suggest that NLRC4 KI mice develop infantile enterocolitis, mirroring the gastrointestinal symptoms observed in AIFEC patients.

Given that human patients develop AIFEC when carrying a heterozygous NLRC4 V341A mutation, we further investigated the phenotype of NLRC4 V341A heterozygous mice. These mice displayed normal growth (Fig. S1A) and only mild intestinal inflammation (Fig. S1B-C). Although heterozygous mice presented slight elevations in serum IL-1β and IL-18 levels during the neonatal period, these levels normalized by 22 days of age (Fig. S1D, E). Notably, the serum ferritin and IL-6 levels remained within the normal range throughout the study period (Fig. S1D-E), indicating minimal systemic autoinflammation. The milder AIFEC pathology observed in infant heterozygous mice may reflect lower levels of inflammasome activation than in homozygous knock-in (KI) mice (Fig. S1F). These findings suggest that the abundance of the mutant allele may play a more critical role in disease pathogenesis in the KI mouse model than in human patients.

Continuous E2a Cre expression in the absence of loxP sites is known to induce a certain degree of DNA damage, which may trigger innate immune responses [24–26]. We therefore evaluated whether Cre expression contributed to the pathogenesis of disease in our E2aCre NLRC4^KI/KI^ mice. By intercrossing E2aCre^+/-^-NLRC4^KI/+^ mice, we generated NLRC4 V341A^KI/KI^ mice lacking E2aCre (hereafter referred to as NLRC4 KI^ΔE2aCre^) along with littermate wild-type controls (NLRC4 WT). Western blot analysis confirmed the expression of the V341A KI allele, which is consistent with E2aCre-mediated germline recombination, which is heritable across generations (Fig. S2A). The NLRC4 KI^ΔE2aCre^ mice displayed markedly reduced growth, with the majority not surviving beyond 10 days after birth (Fig. S2B). They also developed severe autoinflammation (Fig. S2C, D) and enterocolitis with severe diarrhea (Fig. S2E, F), fully recapitulating the phenotype observed in E2aCre–NLRC4^KI/KI^ mice. These findings indicate that the NLRC4 V341A mutation accounts for the pathogenesis of AIFEC in this model, independent of E2a-Cre.

### Gut barrier integrity is impaired in NLRC4 V341A KI mice

**Fig. 4:**
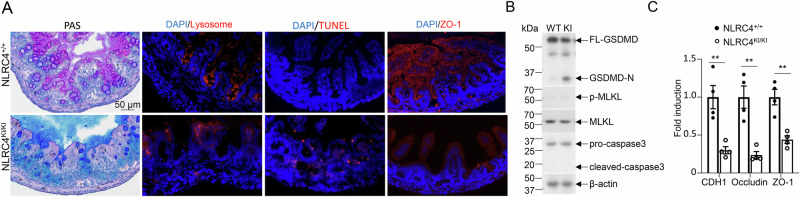
Gut barrier integrity is impaired in NLRC4 V341A KI mice. **A** Representative images of different histochemical stains as indicated; samples were colon (PAS staining) or otherwise distal small intestine tissues from 6-day-old NLRC4 WT and NLRC4 KI mice. Periodic acid–Schiff (PAS) staining was used for goblet cells, lysosomal staining was used for Paneth cells, TUNEL was used for cell death, and ZO-1 was used for tight junction assessment. **B** Western blot analysis of the type of cell death in distal small intestine tissues from 6-day-old NLRC4 WT and NLRC4 KI mice. Cleaved Gasdermin D (GSDMD-N) is indicative of pyroptosis, p-MLKL is a marker of necroptosis, and cleaved caspase 3 signifies apoptotic cell death. **C** Representative tight junction genes, as indicated, were analyzed via Q‒PCR. The samples were intestinal epithelial cells isolated from 6-day-old NLRC4 WT and NLRC4 KI mice (*n* = 4 per group). *p* values were determined by Student’s *t* test, **p < 0.01. The data are shown as the means ± SEMs. The data are representative of three independent experiments

Excessive IL-18 production, as observed in conditions involving the deletion of IL-18BP or colitis, has been linked to the loss of mucus-producing goblet cells [27]. Given that NLRC4 KI mice exhibit overproduction of IL-18 in the gut, we assessed goblet cell populations in the colon tissue via PAS staining to detect mucin-producing goblet cells. This analysis revealed a dramatic reduction in the number of goblet cells in the KI tissue compared with those in the WT control tissue (Fig. 4A, panel 1). Interestingly, we also observed a decrease in Paneth cells in the small intestines of KI mice (Fig. 4A, panel 2). As shown in Fig. 1E, intestinal epithelial cells (IECs) from KI tissue exhibited robust GSDMD cleavage, which is typically associated with pyroptosis. To investigate cell death in the gut tissue, we performed a TUNEL assay, which revealed significant cell death in the KI tissue, whereas almost no death was observed in the WT controls (Fig. 4A Panel 3).

Additionally, Western blot analysis of IECs from both WT and KI mice revealed no differences in necroptosis (assessed by probing for pMLKL) or apoptosis (assessed by probing for cleaved caspase-3) between the two groups (Fig. 4B), suggesting that the increased cell death in the gut epithelium of KI mice is likely due to pyroptosis. Notably, as shown in Fig. 1E, caspase-1 activation was observed in IECs from NLRC4 V341A KI mice but not in those from littermate controls, supporting the role of the caspase-1–GSDMD axis in NLRC4 V341A-induced pyroptosis. Inflammasome activation can also modulate the expression of tight junction proteins via IL-1β or IL-18, and both cytokines have been shown to impair tight junction integrity by inducing downstream inflammatory signaling cascades, including the NF-κB and MAPK pathways, which promote the production of additional cytokines and chemokines and alter the expression or localization of tight junction proteins such as ZO-1 and occludin [28, 29]. We analyzed tight junction proteins in the small intestines of WT and KI mice via immunofluorescence staining for ZO-1, which revealed a dramatic reduction in ZO-1 levels in the KI mice compared with those in the WT controls (Fig. 4A, panel 4). Q‒PCR analysis of CDH1 (cadherin), occludin and ZO-1 further confirmed that the expression levels of these tight junction proteins were significantly lower in the intestinal epithelial cells of the KI mice than in those of the WT controls (Fig. 4C). Collectively, these findings suggest that the integrity of the gut barrier is impaired in NLRC4 KI mice.

### NLRC4 V341A KI mice develop AIFEC after birth

**Fig. 5:**
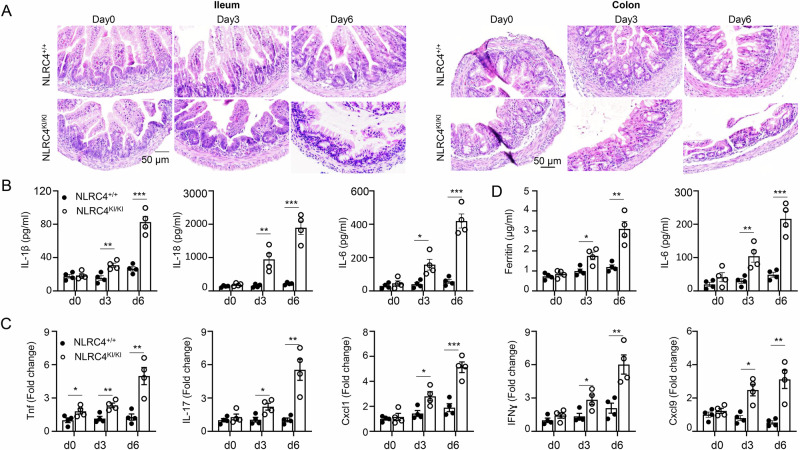
Kinetic analysis of AIFEC development in NLRC4 V341A KI pups. **A** Representative H&E staining of small intestine (left) and colon (right) tissues from 0-, 3-, and 6-day-old NLRC4 WT and NLRC4-KI mice. **B** IL-1β, IL-18 and IL-6 levels in the supernatants of colon explant cultures from NLRC4 WT and KI mice were measured via ELISA. **C** Colon tissues from 0-, 3-, and 6-day-old NLRC4 WT and NLRC4-KI mice were analyzed for inflammatory gene expression as indicated by Q‒PCR. **D** The levels of serum ferritin and IL-6 in 0-, 3-, and 6-day-old NLRC4 WT and NLRC4 KI mice were measured via ELISA. Sample size: *n* = 4/group. The data are shown as the means ± SEMs. *p* values were determined by Student’s *t* test, *p < 0.05, **p < 0.01 and ***p < 0.001. The data are representative of three independent experiments

While we observed severe infantile enterocolitis and autoinflammation in NLRC4-KI mice at 6 days post-natally (Figs. 1–4), it remains unclear whether these mice developed disease earlier, before birth. To investigate this, we monitored disease progression at postnatal days 0, 3, and 6 using littermate wild-type (WT) controls at each time point. Initially, H&E staining of intestinal tissues revealed no significant damage to either the small intestine or colon of NLRC4-KI mice at birth. However, by day 3, we observed crypt disruption and inflammatory infiltrates in the intestines of NLRC4-KI mice, which worsened by day 6 (Fig. 5A).

Consistent with these histological findings, ELISA analysis of supernatants from colon explant cultures revealed no differences in IL-1β, IL-18, or IL-6 levels between the two groups on day 0. However, significant increases were noted in NLRC4 V341A pups at postnatal days 3 and 6 (Fig. 5B). This finding was further supported by qPCR analysis, which revealed elevated expression of proinflammatory cytokines (TNF, IL-17, and IFN-γ) and chemokines (CXCL1 and CXCL9) in KI pups (Fig. 5C). Importantly, qPCR analysis also demonstrated that endogenous NLRC4 expression levels were comparable between WT and KI colon tissues at both day 0 and day 3 (Fig. S3A), indicating that the observed inflammation was not due to differential NLRC4 expression. This finding was corroborated by Western blot analysis (Fig. S3B).

To assess systemic autoinflammation in early infancy, we measured the serum ferritin and IL-6 levels. At birth (day 0), both markers were similar between the KI and WT pups but were significantly elevated in the KI group by postnatal days 3 and 6 (Fig. 5D), indicating the progression of systemic inflammation. Collectively, these data suggest that AIFEC in NLRC4 KI mice arises after birth, with clear pathological and inflammatory changes emerging by postnatal day 3.

### Adult NLRC4 V341A conditional KI mice exhibit autoinflammation with mild colitis

Infant AIFEC patients exhibit severe enterocolitis. In contrast, colitis in adult patients typically resolves or becomes much milder, although autoinflammation persists into adulthood [16, 30]. Constitutive NLRC4-KI pups, however, die within 10 days post-natally, preventing further study of autoinflammation in adult KI mice. To model the adult AIFEC patient phenotype, we employed Rosa26-Cre^ER^ mice [31] to temporally induce global NLRC4 KI expression in adult mice via tamoxifen administration. Eight-week-old Rosa26-Cre^ER^ mice were crossed with NLRC4 V341A^fl/fl^ mice to generate Rosa26-Cre^ER^ NLRC4 V341A^fl/+^ offspring. Further breeding produced Rosa26-Cre^ER^ NLRC4^+/+^ and Rosa26-Cre^ER^ NLRC4 V341A^fl/fl^ mice, which were used for subsequent experiments. To generate conditional NLRC4 KI (cKI) mice, 6-week-old Rosa26-Cre^ER^ NLRC4 V341A^fl/fl^ mice were treated with tamoxifen for five consecutive days. Littermate Rosa26-Cre^ER^ NLRC4^+/+^ mice treated with tamoxifen served as controls. Mouse body weight and survival were monitored daily following tamoxifen treatment. Compared with control mice, NLRC4 cKI mice exhibited normal survival and no significant changes in body weight (data not shown). At 8 weeks of age, both NLRC4 cKI and control mice were euthanized for genotyping and phenotypic analysis. Western blot analysis of small intestine tissue and intestinal epithelial cells (IECs) confirmed NLRC4 knock-in expression in the NLRC4 cKI mice (Fig. S4A).

Phenotypically, NLRC4 cKI mice presented splenomegaly and hepatomegaly (Fig. S4B). Blood analysis revealed cytopenia, as indicated by reduced blood cell counts (Fig. S4C). The serum levels of ferritin, AST, IL-1β, IL-18, and IL-6 were significantly elevated in NLRC4 cKI mice compared with those in controls (Fig. S4D-E), whereas the hemoglobin levels were markedly reduced, further supporting the presence of robust autoinflammation in these mice (Fig. S4D). To assess intestinal inflammation, colon explant cultures were performed, revealing a significant increase in IL-1β, IL-18, and IL-6 levels in the supernatants of NLRC4 cKI mice compared with tamoxifen-treated controls (Fig. S4F). qPCR also revealed upregulated expression of inflammatory cytokines and chemokines in colon tissues from NLRC4 cKI mice (data not shown). However, despite these inflammatory markers, we did not observe substantial damage or inflammatory infiltrates in the gut epithelium of adult NLRC4 cKI mice, which contrasts with the severe epithelial damage observed in infantile NLRC4 KI mice (Fig. S4G). Additionally, no significant differences were observed in stool scores between NLRC4 cKI mice and WT controls (Fig. S4H). Together, these findings suggest that adult NLRC4 cKI mice exhibit autoinflammation with mild colitis, serving as a relevant model for adult AIFEC patients.

### IL-18 blockade improves survival in NLRC4 V341A KI pups with AIFEC

Previous studies have shown that IL-18 blockade is effective in treating AIFEC patients [32, 33]. However, these treatments were either preceded by other medications or combined with IL-1β blockade, complicating data interpretation. To eliminate these confounding factors, evaluating the therapeutic effects of IL-18 blockade in isolation is essential. Our novel animal model for AIFEC provides a unique platform to address this question. We treated pups with recombinant IL-18BP beginning on day 1 after birth and administered it consecutively for 7 days. The treated KI pups appeared significantly healthier than the untreated controls did by the end of the treatment period. To further assess the safety of treatment withdrawal, we discontinued therapy after 7 days and monitored the pups for another 2 weeks.

**Fig. 6:**
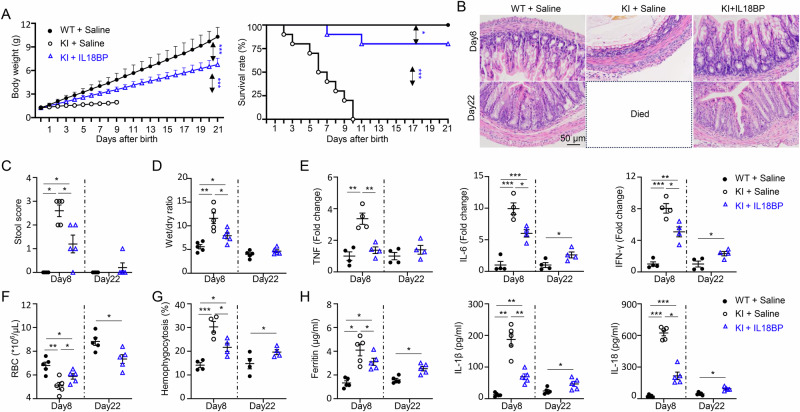
IL18BP treatment improves survival in NLRC4 V341A KI pups with AIFEC. IL-18BP was administered to NLRC4 V341A KI pups via intraperitoneal (i.p.) injection at a dose of 8 mg/kg from day 1 to day 7 after birth. The NLRC4 WT (WT) and KI control groups received equivalent volumes of saline. Pups were monitored daily for weight and survival. **A** Body weights and survival rates of the indicated groups. **B** Representative H&E staining of colon tissues from 8-day-old and 22-day-old mice as indicated. **C** Stool scores of the indicated groups of mice on days 8 and 22 posttreatment. **D** Wet-to-dry feces ratios in the indicated groups. **E** Inflammatory gene expression in colon tissues from 8-day-old and 22-day-old mice was analyzed by real-time PCR. **F** Red blood cell (RBC) counts in blood samples from 8-day-old and 22-day-old mice. **G** Hemophagocytosis in the spleen was analyzed by flow cytometry, which revealed the percentage of myeloid cells that phagocytosed RBCs. **H** Serum levels of ferritin, IL-1β, and IL-18 in 8-day-old and 22-day-old mice were measured via ELISA. The data are presented as the means ± SEMs. Sample size: *n* = 5/group, except for panels E&G (*n* = 4/group). Statistical significance was determined via Student’s *t* test (*p < 0.05, **p < 0.01, ***p < 0.001). The results are representative of at least two independent experiments

Our findings revealed that a 7-day IL-18BP treatment significantly enhanced the growth of NLRC4 KI pups, with body weights reaching 65.6% of those of WT pups by day 22, and improved survival rates to 80% compared with those of untreated KI mice, which died within 10 days after birth (Fig. 6A). H&E staining of colon tissue on day 8 revealed that IL-18BP treatment effectively reduced gut epithelial disruption in NLRC4-KI pups (Fig. 6B). Notably, epithelial integrity remained stable even after drug withdrawal, as indicated by H&E staining on day 22 (Fig. 6B). Consistently, IL-18 blockade improved the stool score (Fig. 6C) and reduced diarrhea, as evidenced by a lower wet‒dry/feces ratio on day 8 posttreatment. By day 22, no differences in stool scores or fecal properties were observed between treated KI mice and WT controls (Fig. 6D). IL-18BP treatment also suppressed the expression of proinflammatory cytokines (TNF, IL-6, and IFN-γ) (Fig. 6E) and chemokines (CXCL9 and CXCL10, data not shown) on day 8, underscoring its anti-inflammatory effects. However, elevated levels of IL-6 and IFN-γ persisted in the gut epithelium of treated KI mice compared with WT controls on day 22, indicating that complete homeostasis had not been restored (Fig. 6E). Together, these results demonstrate that colitis in KI mice was significantly ameliorated following IL-18 blockade and continued to improve over time, even after drug withdrawal (Fig. 6B-E).

AIFEC patients also exhibit systemic autoinflammation in addition to infantile colitis. Notably, compared with no treatment, IL-18BP treatment alleviated MAS, as evidenced by improved cytopenia (Fig. 6F), reduced hemophagocytosis (Fig. 6G), and decreased serum levels of ferritin, IL-1β, and IL-18 on day 8 (Fig. 6H). Despite these improvements, residual autoinflammation persisted relative to that in WT controls at both day 8 and day 22, although it progressively diminished with age (Fig. 6F-H). Collectively, these findings highlight that IL-18 blockade mitigates infantile enterocolitis and attenuates autoinflammation in NLRC4 KI mice, significantly improving survival outcomes.

### TNF blockade dramatically alleviates disease in the human-relevant AIFEC model

Very early-onset IBD (VEO-IBD), defined as disease onset before 6 years, is more heterogeneous and often life-threatening than later-onset IBD is [34]. Infliximab, a chimeric monoclonal IgG1 antibody against tumor necrosis factor (TNF), was the first biologic approved for the treatment of moderate to severe inflammatory bowel disease (IBD) in adults over 20 years ago [35, 36]. Consequently, most VEO-IBD patients are treated off-label with conventional TNF antagonists. Notably, a recent comprehensive study of 216 VEO-IBD patients demonstrated that the efficacy and safety of TNF blockade in this population are comparable to those reported in older IBD patients, supporting its continued use in treating VEO-IBD [37].

**Fig. 7:**
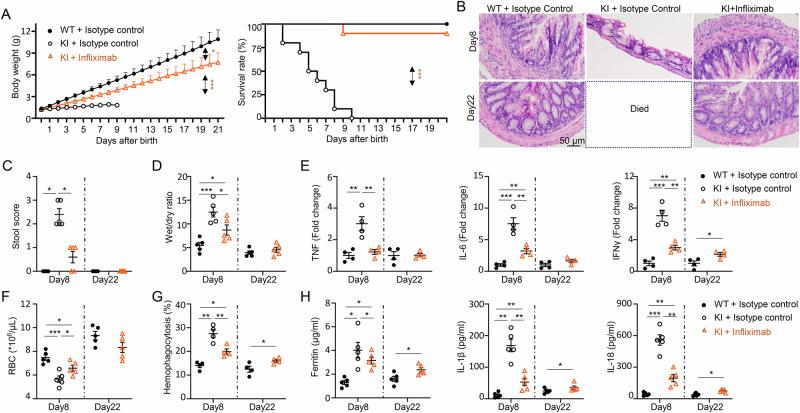
TNFα blockade dramatically alleviates AIFEC in NLRC4 V341A KI pups. Infliximab was administered to NLRC4 V341A KI pups via intraperitoneal (i.p.) injection at a dose of 5 mg/kg from day 1 to day 7 after birth. The control groups received equivalent volumes of isotype control antibodies. Pups were monitored daily for weight and survival. **A** Body weights and survival rates of the indicated groups. **B** Representative H&E staining of colon tissues from 8-day-old and 22-day-old mice as indicated. **C** Stool scores of the indicated groups of mice on days 8 and 22 posttreatment. **D** Wet-to-dry feces ratios in the indicated groups. **E** Inflammatory gene expression in colon tissues from 8-day-old and 22-day-old mice was analyzed by real-time PCR. **F** Red blood cell (RBC) counts in blood samples from 8-day-old and 22-day-old mice. **G** Hemophagocytosis in the spleen was analyzed by flow cytometry, which revealed the percentage of myeloid cells that phagocytosed RBCs. **H** Serum levels of ferritin, IL-1β, and IL-18 in 8-day-old and 22-day-old mice were measured via ELISA. The data are presented as the means ± SEMs. Sample size: *n* = 5/group, except for panels E&G (*n* = 4/group). Statistical significance was determined via Student’s *t* test (*p < 0.05, **p < 0.01, ***p < 0.001). The results are representative of at least two independent experiments

NLRC4-mediated infantile enterocolitis, a subtype of VEO-IBD, typically manifests before the age of 2 [16, 38]. On the basis of these findings, we hypothesized that TNF blockade would be effective in treating AIFEC. To test this hypothesis, we evaluated the effects of TNF-α antibody treatment in our novel AIFEC animal model. Seven-day infliximab treatment, administered via intraperitoneal injection from day 1 to day 7 postnatally, significantly improved the growth of NLRC4 KI pups, with body weights reaching 70.7% of those of WT controls by day 22 (Fig. 7A, left) and survival rates increasing to 90% (Fig. 7A, right). TNF-α antibody treatment effectively protected NLRC4-KI pups from epithelial damage (Fig. 7B) and significantly alleviated colitis by day 8, with near-complete resolution by day 22. Evidence of this includes reduced stool scores (Fig. 7C), improved diarrhea (Fig. 7D), and decreased expression of proinflammatory genes, such as TNF, IL-6, and IFN-γ (Fig. 7E). Moreover, TNF-α antibody treatment mitigated systemic autoinflammation in KI mice, as evidenced by increased blood cell counts (Fig. 7F), decreased hemophagocytosis (Fig. 7G), and reduced systemic inflammation (Fig. 7H). Collectively, these findings demonstrate that TNFα neutralization with infliximab mitigates infantile enterocolitis, attenuates autoinflammation, and significantly improves survival in NLRC4 KI mice.

### Glucose supplementation unexpectedly protects NLRC4 V341A KI pups from AIFEC

Biochemical analysis revealed that NLRC4-KI pups presented significantly lower blood glucose levels than WT controls did (Fig. 2D). Using the Accu-Chek Glucose Monitor Kit, we further confirmed this finding: glucose levels in KI pups ranged from 18 to 29 mg/dL on day 6, whereas they ranged from 129 to 158 mg/dL in WT pups. These data collectively indicate that NLRC4 KI mice developed severe hypoglycemia. Hypoglycemia in neonates often requires intensive care and is associated with increased mortality in the neonatal ICU [39–41]. Glucose supplementation is a standard treatment for hypoglycemia in children [42]. On the basis of these observations, we hypothesized that glucose supplementation could have therapeutic effects on the AIFEC model.

**Fig. 8:**
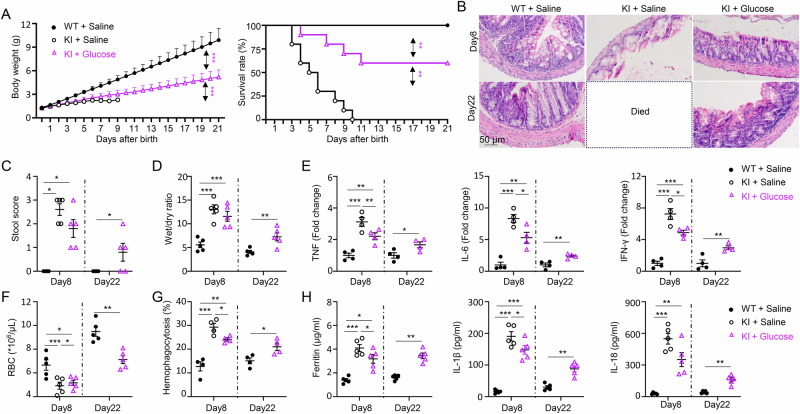
Glucose supplementation protects NLRC4 V341A KI pups from AIFEC. Glucose was administered to NLRC4 V341A KI pups via intraperitoneal (i.p.) injection at a dose of 200 mg/kg from day 1 to day 7 after birth. The control groups received equivalent volumes of saline. Pups were monitored daily for weight and survival. **A** Body weights and survival rates of the indicated groups. **B** Representative H&E staining of colon tissues from 8-day-old and 22-day-old mice as indicated. **C** Stool scores of the indicated groups of mice on days 8 and 22 posttreatment. **D** Wet-to-dry feces ratios in the indicated groups. **E** Inflammatory gene expression in colon tissues from 8-day-old and 22-day-old mice was analyzed by real-time PCR. **F** Red blood cell (RBC) counts in blood samples from 8-day-old and 22-day-old mice. **G** Hemophagocytosis in the spleen was analyzed by flow cytometry, which revealed the percentage of myeloid cells that phagocytosed RBCs. **H** Serum levels of ferritin, IL-1β, and IL-18 in 8-day-old and 22-day-old mice were measured via ELISA. The data are presented as the means ± SEMs. Sample size: *n* = 5/group, except for (**E,** **G**) (*n* = 4/group). Statistical significance was determined via Student’s *t* test (*p < 0.05, **p < 0.01, ***p < 0.001). The results are representative of at least two independent experiments

To test this hypothesis, we administered glucose to KI pups via intraperitoneal injection for 7 days post-natally (days 1–7). This regimen significantly improved survival, increasing the survival rate of KI pups to 60% (Fig. 8A, right). Additionally, glucose supplementation partially ameliorated growth retardation, with treated pups reaching 52.6% of the WT body weight at two weeks post-treatment (Fig. 8A, left). Histological analysis revealed that glucose supplementation reduced colitis, as shown by improved colon epithelial integrity on days 8 and 22 posttreatment (Fig. 8B). These findings were corroborated by reduced stool scores (Fig. 8C), increased wet/dry feces ratios (Fig. 8D), and decreased expression of proinflammatory genes, including *TNF*, *IL-6*, and *IFN-γ* (Fig. 8E).

Moreover, glucose supplementation attenuated MAS in KI mice. Treated pups presented increased blood cell counts (Fig. 8F), decreased hemophagocytosis (Fig. 8G), and reduced ferritin levels (Fig. 8H). Collectively, these results highlight glucose supplementation as a promising nutritional intervention to mitigate disease severity in AIFEC, providing a basis for further exploration in clinical contexts.

## Discussion

While NLRC4 inflammasome activation is critical for host defense, excessive NLRC4 inflammasome activation can lead to severe disease, as evidenced by decreased survival, severe intestinal bleeding and multiple-organ damage induced by *E. coli* O21:H^+^-induced NLRC4 inflammasome overactivation [43]. This phenotype was recapitulated by systemic activation of the NLRC4 inflammasome by injecting mice with bacterial needle/rod proteins [44]. Furthermore, eicosanoids produced upon NLRC4 inflammasome activation have been shown to contribute to enterocolitis [44]. However, direct evidence linking excessive NLRC4 inflammasome activation to human disease comes from two pioneering reports in 2014, which identified gain-of-function mutations in NLRC4 (specifically T337S and V341 A) as contributors to the pathogenesis of autoinflammatory enterocolitis (AIFEC) [10, 11]. Since then, the spectrum of associated mutations has expanded [12–15]. In this study, we present a conditional NLRC4 V341A KI mouse model in which pups spontaneously develop severe infantile enterocolitis and autoinflammation. In contrast, adult conditional KI mice predominantly exhibit autoinflammation with only mild enterocolitis. AIFEC is characterized by life-threatening but usually transient enterocolitis during infancy and recurrent, severe autoinflammation throughout life [8, 16]. Our NLRC4 KI model effectively recapitulates these key features, with infant mice succumbing within 10 days post-birth, closely mirroring the high mortality rates observed in infant AIFEC patients, many of whom do not survive beyond early childhood without appropriate medical intervention. These findings underscore the importance of our novel animal model for studying AIFEC.

Interestingly, two independent studies by Eeckhout et al. [45] and Weiss et al. [19]. reported NLRC4 V341A and NRLC4 T337S knock-in mouse models that did not develop the AIFEC phenotype, although both exhibited elevated IL-18 levels. The mechanisms underlying these divergent outcomes remain incompletely understood, but several methodological and contextual distinctions may help explain why our NLRC4 V341A model manifests spontaneous autoinflammation whereas the prior models did not. First, our model uses a Cre-inducible knock-in strategy in which mutant Nlrc4 cDNA is inserted into the endogenous locus behind a loxP–stop–loxP cassette, allowing conditional expression only after Cre-mediated recombination. In contrast, the two prior models employed CRISPR/Cas9-mediated genome editing to introduce the mutation directly into the native Nlrc4 coding sequence. Notably, our Nlrc4V341A construct carries three Flag tags, whereas the constructs used by Weiss et al. and Eeckhout et al. do not. This makes the protein larger than wild-type NLRC4. Although Flag tags are commonly used for detection, we cannot exclude the possibility that they may have contributed to the spontaneous inflammasome activity observed in our study. This phenomenon should be further addressed in the future. Second, while all three models—NLRC4 V341A [45], NLRC4 T337S [19], and our model—display elevated serum IL-18, only our model develops spontaneous autoinflammation, indicating that IL-18 elevation alone is not sufficient to drive disease. Notably, Weiss et al. demonstrated that IL-18 production in their model was ASC dependent, confirming inflammasome activation. The presence of autoinflammation in our system may reflect a greater magnitude of inflammasome activation, contributions from additional downstream effectors such as IL-1β or GSDMD in addition to IL-18, or cofactors—such as TNF signaling—that we found to be critical in our model. Third, environmental influences, including facility-specific differences in housing or microbiota composition, may also contribute to variability in disease penetrance and severity.

In our study, the observed postnatal growth retardation in NLRC4 V341A KI mice coincided temporally with the onset of systemic inflammation and intestinal pathology. Notably, this phenotype mirrors the failure to thrive reported in patients harboring NLRC4 gain-of-function mutations. On the basis of the temporal correlation and elevated inflammatory cytokine profiles, we interpret the reduced growth as a secondary consequence of systemic inflammation, likely mediated by sustained IL-1β and IL-18 signaling and their downstream catabolic effects. This finding is consistent with clinical observations in autoinflammatory syndromes, where chronic inflammation disrupts normal growth trajectories [10, 11, 46]. In addition to prolonged cytokine overproduction, hyperactivation of the NLRC4 inflammasome may also interfere with the growth hormone (GH)–IGF-1 axis, impair nutrient absorption, reduce energy availability, or directly affect bone and cartilage development [47, 48]. However, the exact mechanisms remain to be determined and warrant further investigation.

Colon shortening is a commonly used gross indicator of colitis severity. However, in our study, we did not observe consistent or significant shortening of the colon in the NLRC4 V341A KI mice at the examined timepoint. Interestingly, in some cases, the colons appeared slightly longer, which may reflect early postnatal intestinal growth dynamics or compensatory elongation due to epithelial remodeling rather than classic fibrotic contraction. We believe this observation is likely due to the timing of disease onset, age-specific tissue responses, and/or the unique pathogenesis associated with NLRC4 inflammasome activation. These features distinguish our model from conventional adult-onset colitis models (e.g., DSS or T-cell transfer), in which colon shortening is more consistently observed.

Given that AIFEC is a rare and often severe condition, therapeutic exploration in clinical settings remains challenging. Our NLRC4 KI mouse model provides a unique platform to bridge this gap and facilitate preclinical studies. Using this model, we assessed the therapeutic efficacy of several potential AIFEC treatments. Recombinant IL-18 binding protein (IL-18BP) has been employed in treating AIFEC but has been tested in only a limited number of cases, often in combination with other anti-inflammatory agents [32, 33], complicating the interpretation of its efficacy. In our study, IL-18BP treatment significantly improved survival, growth, and colitis in KI pups while also reducing systemic autoinflammation and suppressing proinflammatory cytokines. These findings are the first to demonstrate that IL-18 blockade alone can prevent disease progression in AIFEC. We also explored TNF blockade, a treatment known for its efficacy in inflammatory bowel disease (IBD) [35–37]. Interestingly, TNF inhibition dramatically improved survival rates (90%) and alleviated both colitis and systemic inflammation in our preclinical AIFEC model. A prior report revealed no benefit from TNF blockade in a single AIFEC patient [32], possibly due to treatment initiation at an advanced disease stage and a limited number of patient cases, whereas our study involved intervention at the earliest stages.

Additionally, we investigated the therapeutic role of glucose supplementation, given the severe hypoglycemia observed in KI pups. Glucose supplementation significantly improved survival and reduced disease severity, highlighting the need for glucose monitoring and timely intervention in AIFEC patients. These results suggest that glucose supplementation may serve as an effective adjunct therapy in the early stages of the disease, although its underlying mechanisms remain to be fully clarified. We propose three interconnected pathways through which glucose may exert its effects. First, glucose supports immune cell function by serving as a vital energy source during periods of heightened activation [49, 50], such as systemic inflammation. Inflammatory states increase immune cell metabolic demands, and glucose supplementation may help maintain immune balance and reduce excessive inflammatory responses, as supported by studies showing reduced white blood cell counts and atherosclerotic lesions in glucose-treated, high-fat diet-fed mice [51]. Second, glucose appears to enhance intestinal epithelial barrier integrity by promoting the expression and assembly of tight junction proteins such as claudins and occludins [52]. Both glucose deficiency and excess glucose are known to impair this barrier and exacerbate inflammation [53, 54], whereas optimal glucose levels help maintain barrier function and limit antigen translocation. Third, glucose can modulate the gut microbiota, encouraging the production of anti-inflammatory microbial metabolites that shape mucosal immune responses [55, 56]. Overall, these findings suggest that glucose supplementation may alleviate disease in NLRC4 V341A KI mice by restoring immune metabolic balance, strengthening epithelial barriers, and promoting a beneficial microbial environment. Further metabolic profiling is needed to validate these proposed mechanisms.

Very early-onset inflammatory bowel disease (VEO-IBD), which is diagnosed in individuals under the age of six, is a heterogeneous and severe disorder with potentially fatal outcomes. The incidence of VEO-IBD is increasing, but the number of approved therapies remains limited, and traditional treatments for IBD often prove ineffective [37, 57]. Over 75 distinct single-gene defects have been identified as molecular causes of VEO-IBD, accounting for approximately 7.85–7.9% of cases [34, 37, 58]. The disease is influenced by a complex interplay of environmental factors, immune dysregulation, impaired gut barrier function, and dysbiosis in genetically susceptible individuals [59]. Several of these monogenic defects have been associated with dysregulated inflammasome activity, underscoring the central role of inflammasomes in maintaining intestinal homeostasis [38]. The discovery of de novo gain-of-function mutations in NLRC4, which lead to infantile enterocolitis in patients, established a direct connection between VEO-IBD pathogenesis and dysregulated inflammasome activation [11, 38, 58, 60]. Our NLRC4 V341A KI mouse model thus offers a valuable tool for studying the pathogenesis of monogenic VEO-IBD. The model exhibits hallmark features of infantile enterocolitis, including gut inflammation, impaired intestinal barrier integrity, disrupted epithelium, and severe diarrhea. These findings position our model as a unique resource for dissecting the disease mechanisms of VEO-IBD and testing novel therapeutic approaches.

In summary, our study demonstrated the therapeutic potential of IL-18 blockade, TNF inhibition, and glucose supplementation in AIFEC. As most AIFEC patients are severely ill at diagnosis, future studies should evaluate these interventions in later disease stages to refine treatment strategies. Collectively, our findings establish the NLRC4 V341A KI mouse model as a robust platform for advancing our understanding of AIFEC, VEO-IBD, and related autoinflammatory diseases while providing a basis for developing targeted therapies.

## Materials and methods

### Animals

The NLRC4 V341A-Flag^flox/flox^ mice were generated by Cyagen Biosciences via Cyagen’s TurboKnockout^®^ gene targeting service through homologous recombination. The Nlrc4 gene (NCBI reference sequence: NM_001033367.3) is located on mouse chromosome 17. Nine exons have been identified, with the ATG start codon in exon 2 and the TAA stop codon in exon 9. In the targeting vector, the coding sequence of exon 2 was replaced with the “loxP-stop-loxP kozak-mutant Nlrc4 CDS-3*Flag-IRES-EGFP-polyA”. The V341A (GTG to GCG) mutation was introduced into the mutant Nlrc4 CDS. In the targeting vector, the Neo cassette was flanked by self-deletion anchor (SDA) sites. Diphtheria toxin A (DTA) was used for negative selection (Fig. 1A). C57BL/6 ES cells were used for gene targeting. The final targeting vector was sequenced for validation. The NLRC4 V341A-Flag^flox/flox^ mice were genotyped via PCR and further confirmed by sequencing. After the Cre transgenic mice were bred in our facility, western blotting was conducted to verify their expression at the protein level via the use of a Flag tag antibody and NLRC4 antibody. E2a-Cre (JAX, #003724) [21] and R26-CreERT2 (JAX,008463) [31] mice were purchased from the Jackson Laboratory. Both male and female mice were used. Littermate controls were used for all the experiments. The mouse ages are indicated in the figure legends. All the mice were bred and maintained in individually ventilated cages under specific pathogen-free conditions in accredited animal facilities. The animal experiments were approved by the Institutional Animal Care and Use Committee of the University of Iowa.

### Culture of bone marrow-derived macrophages (BMDMs)

Bone marrow was harvested from the femurs of the mice by flushing with RPMI-1640 medium containing 2% FBS. The harvested cells were centrifuged at 300 × *g* for 10 min, followed by treatment with ACK lysis buffer (Thermo Fisher, A1049201) for 30 s to lyse red blood cells. To neutralize the lysis buffer, 10 volumes of PBS were added, and the cells were centrifuged again at 300 × *g* for 10 min. The resulting cell pellet was resuspended in BMDM culture medium (RPMI-1640 supplemented with 10% FBS, antibiotics, L-glutamine, and 10 ng/ml M-CSF) and plated at a density of 1 × 10^6^ cells/ml in tissue culture plates. The culture medium was changed on days 4 and 6, and the BMDMs were used on day 7.

### Colonic explant culture

The whole colons were harvested, thoroughly rinsed with serum-free DMEM, and weighed to determine their initial weight. The collected colon tissues were cut into 2 mm pieces, cultured as explants in regular RPMI 1640 medium supplemented with 10% FBS, L-glutamine, penicillin, and streptomycin, and placed in a standard cell culture incubator for 24 h. After culture, the cell-free supernatants were obtained via centrifugation at 12,000 × *g* for 10 min at 4 °C and stored in aliquots at −20 °C for further analysis.

### Blood chemical parameters assay

The chemical parameters of blood from NLRC4 WT and KI mice were measured as indicated (ALT, alanine aminotransferase; BUN, blood urea nitrogen; LDH, lactate dehydrogenase, etc.) by service from IDEXX, Inc., via a VetScan VS2 analyzer.

### ELISA analysis

The serum levels of ferritin, AST, and hemoglobin, as well as those of IL-6, IL-1β, and IL-18, in both the serum and colon explant cultures were quantified via commercial ELISA kits according to the manufacturer’s instructions. The kits used were Ferritin (catalog #80636, Crystal Chem), AST (catalog #XPEM0857, Xpress Bio), hemoglobin (catalog #80640, Crystal Chem), IL-1β (catalog #DY401-050, R&D), IL-6 (catalog #DY406-05, R&D), and IL-18 (catalog #7625-05, R&D). The cytokine levels in the colon explant supernatants were normalized to the weight of the colon tissue.

### Peripheral blood cell count via a hemocytometer

Peripheral blood samples were collected from the NLRC4 WT and KI mice in EDTA-coated tubes to prevent coagulation. Peripheral blood cell counts were performed via a hemocytometer following methods established in previous studies [61].

#### Red blood cell (RBC) counting

Blood samples were diluted with an RBC dilution solution (Hayem’s solution) consisting of mercuric chloride (0.5 g), sodium chloride (1 g), sodium sulfate (5 g), and distilled water (200 mL). A clean RBC pipette was used to draw blood to a mark of 0.5, followed by filling the pipette to mark 101 with the RBC diluting solution, creating a 1:200 dilution. The mixture was thoroughly mixed, and the first few drops were discarded. The diluted sample was then loaded into a hemocytometer, and the cells were allowed to settle. RBCs in the central large square were counted under a microscope at 40× magnification. The total RBC concentration was calculated via the standard hemocytometer formula, accounting for dilution, area, and chamber depth.

#### Platelet counting

A platelet dilution solution of 1% ammonium oxalate was prepared by dissolving 1 g of ammonium oxalate in 100 mL of distilled water. The RBC pipette was precleaned with alcohol before blood was drawn to a mark of 0.5, and the pipette was then filled to mark 101 with the platelet dilution solution, resulting in a 1:200 dilution. After the sample was thoroughly mixed, the first few drops were discarded, and the diluted blood was loaded into the hemocytometer. Platelets in the central large square were counted under a microscope at 10x magnification, and the total platelet concentration was calculated via the standard hemocytometer formula.

#### White blood cell (WBC) counting

WBC counting was performed via a hemocytometer, a WBC pipette, and a WBC dilution mixture composed of glacial acetic acid (1.5 mL), crystal violet solution (1% aqueous, 1 mL), and distilled water (98 mL). The WBC pipette was cleaned with alcohol, and 0.5% blood was drawn. After removing any excess blood from the outside of the pipette, the pipette was filled to mark 11 with the WBC dilution solution, resulting in a 1:20 dilution. The mixture was then thoroughly mixed, and the first drops were discarded before the diluted sample was loaded into the hemocytometer. WBCs were counted in all four corner large squares under a microscope at 10× magnification, and the total WBC concentration was calculated via the standard hemocytometer formula.

### Isolation of intestinal epithelial cells (IECs)

Intestinal epithelial cells (IECs) were isolated following a standard method as previously described [62]. The large intestine was carefully removed, and any attached tissue was trimmed away. The intestine, excluding the cecum, was opened lengthwise and cut into 1 cm pieces, which were then rinsed three times with ice-cold PBS. These pieces were further cut into 5 mm segments and placed in a solution of 5 mM EDTA/PBS at 4 °C on a rocking platform for 30 min. The crypts were then released by shaking the tubes for 2 min and collected by spinning at 200 × *g* for 10 min at 4 °C.

### Isolation of macrophages from the spleen

Macrophages were isolated from mouse spleens via a standardized protocol to ensure high purity and viability. Spleens were carefully harvested from the mice and placed in cold PBS. The spleen tissue was homogenized by gently pressing it through a 70 μm cell strainer via the plunger of a syringe. The cell suspension was then centrifuged at 300 × *g* for 5 min at 4 °C to pellet the cells. Red blood cells were lysed with ACK lysis buffer for 5 min at room temperature, followed by another round of centrifugation and washing with cold PBS. After washing, the cell suspension was enriched for macrophages via magnetic cell separation (MACS) technology. F4/80^+^ macrophages were positively selected via microbeads (catalog #130–110–443; Miltenyi Biotec) according to the manufacturer’s instructions. The purified macrophages were then lysed in TRIzol reagent (Invitrogen) for RNA extraction and Q-PCR.

### Isolation of lamina propria cells

Lamina propria cells were isolated via a modified version of a previously described protocol [63]. Briefly, large intestines, which were cleared of Peyer’s patches, were gently removed from the abdominal cavity. The mesentery and associated fatty tissue were carefully excised via forceps. The intestines were then opened longitudinally and cut into 1 cm segments. These segments were thoroughly washed with room temperature PBS three times. After washing, the intestinal pieces were incubated in a solution of PBS plus 30 mM EDTA and 10 mM HEPES at 37 °C on a rocking platform for 10 min. The epithelial cell layer was removed by shaking the tissue for 2 min. The remaining intestinal fragments were further cut into 5 mm pieces and digested in a solution containing collagenase VIII (200 U/ml, Sigma-Aldrich) and DNase I (150 μg/ml, Sigma-Aldrich) in RPMI 1640 complete medium for 50 min. Following digestion, the cells were resuspended in 4 ml of 40% Percoll (GE Healthcare) and layered onto 2.5 ml of 80% Percoll in a 15 ml tube. The cells were collected from the interphase of the Percoll gradient, washed twice, and resuspended in PBS with 1% FBS. The isolated lamina propria cells were then prepared for flow cytometry analysis.

### Colon diameter measurement

To measure the colon diameter, distal colon sections were fixed, embedded in paraffin, sectioned, and examined under a microscope via calibrated imaging software to determine the external diameter. Multiple measurements were obtained per section, and the average diameter was calculated and recorded.

### Fecal wet/dry ratio

The fecal wet/dry ratio was determined via a modified version of a previously described protocol [64]. Briefly, fecal samples were collected from the contents of the small intestine and colon. Each sample was initially weighed to obtain the wet weight (WW) and then dried in a 70 °C incubator until a constant weight was achieved. The dried samples were subsequently weighed to determine the dry weight (DW). The wet/dry ratio was calculated by dividing the initial wet weight by the final dry weight (WW/DW).

### Flow cytometry

The isolated lamina propria cells were resuspended in PBS containing 5% FBS and stained with fluorescence-conjugated antibodies against CD4 (APC-cy7-CD4, Catalog#100526, clone RM4-5), CD8 (APC-CD8, Catalog# 100712, clone 53-6.7, Biolegend), CD45 (Percp-CD45, Catalog# 103129, clone 30-F11), Ly6G (APC-Ly6G, Catalog# 127613, clone 1A8), F4/80 (PE-F4/80, Catalog# MCA497, clone Cl:A3-1, Bio-Rad) and isotype controls purchased from BD Biosciences. The antibodies were diluted 1:100 when used. Hemophagocytosis in the spleen was analyzed by flow cytometry. Splenocytes isolated from 6-day-old NLRC4 wild-type (WT) and knock-in (KI) mice were initially stained with fluorescence-conjugated antibodies targeting CD11b (percp-CD11b, BioLegend, Catalog#101230, clone M1/70). After surface staining, the cells were incubated with an unconjugated Ter119 antibody (catalog #116202, BioLegend) to block the respective surface antigens. The cells were then permeabilized to allow intracellular staining for phagocytosed Ter119 (FITC-Ter119, Catalog#116205, BioLegend) cells. Flow cytometry was performed using a Cytek Aurora, and the data were analyzed with FlowJo software.

### Immunoblot

Colon tissues or BMDMs were lysed via radioimmunoprecipitation (RIPA) assay buffer supplemented with Complete Mini Protease Inhibitor Cocktail and Phosphatase Inhibitor Cocktail from Roche. The lysates were placed on ice for 30 min and vortexed every 5 min. Following centrifugation at 13,000 rpm for 10 min at 4 °C, the supernatants were collected. The protein concentration was determined with a BCA protein assay kit from Pierce. The proteins were subsequently resolved via SDS‒PAGE and transferred to a 0.45-mm PVDF membrane. For immunoblot analysis, the specified primary antibodies were used at a 1:1000 dilution. The primary antibodies used were as follows: anti-caspase-1 antibody (catalog #AG-20B-0042-C100, AdipoGen); anti-GSDMD antibody (catalog #ab209845, Abcam); anti-mouse IL-1β antibody (catalog #AF-401-NA, R&D); anti-β-actin antibody (catalog #3700S, CST); anti-NLRC4 antibody (catalog #061125, Sigma‒Aldrich); anti-Flag antibody (catalog #F1804, Sigma‒Aldrich); p-MLKL (catalog #91689S, CST); MLKL (catalog #26539T, CST); and anti-caspase-3 antibody (catalog #9661, CST). Horseradish peroxidase (HRP)-conjugated secondary antibodies were selected according to the host species of the primary antibodies. The membranes were treated with enhanced chemiluminescence (ECL) reagent (catalog #34580, Thermo Fisher Scientific), and signal detection was performed via exposure to X-ray film (Z&Z Medical, Fuji X-ray Film).

### Quantitative PCR

Colon tissues or isolated macrophages from the spleen were carefully collected and homogenized via TRIzol reagent (Invitrogen) to ensure efficient RNA extraction. RNA was isolated according to the manufacturer’s instructions. High-quality RNA (500 ng) was then reverse transcribed into complementary DNA (cDNA) via a high-capacity cDNA reverse transcription kit (Applied Biosystems). Quantitative PCR (Q-PCR) was subsequently carried out via SYBR Green Real-time PCR Master Mix (catalog #K1070, APExBio) on a real-time PCR system (Applied Biosystems). Each reaction was performed in triplicate in a 10 μl volume containing 5 μl of SYBR Green mix, 0.3 μM forward and reverse primers, and 0.5 μl of cDNA. The relative gene expression levels were calculated via the comparative Ct (ΔΔCt) method and normalized to the expression of the housekeeping gene β-actin.

### Immunohistochemistry

Formalin-fixed, paraffin-embedded distal small intestine tissue sections were carefully processed for immunohistochemistry (IHC). First, the sections were deparaffinized by immersion in xylene (3 changes, 5 min each), rehydrated through a graded ethanol series (100%, 95%, 70%, and 50%) and finally rinsed with distilled water. To unmask the antigens, antigen retrieval was performed by heating the sections in citrate antigen retrieval buffer (pH 6.0) at 95°C for 30 min in a pressure cooker, followed by gradual cooling to room temperature. After cooling, the tissue sections were permeabilized with 0.1% Triton X-100 in PBS for 10 min, and nonspecific binding sites were blocked with 5% normal goat serum in PBS for 1 h at room temperature. The sections were then incubated overnight at 4 °C with primary antibodies, including anti-lysozyme (catalog #sc-518012, Santa Cruz Biotechnology, 1:500 dilution) and anti-ZO-1 (catalog #14–9776–82, eBioscience, 1:500 dilution) antibodies. Control sections were incubated with isotype-matched IgG antibodies. The following day, the sections were washed three times with PBS and incubated for 1 h at room temperature with fluorescence-conjugated secondary antibodies. After secondary antibody incubation, the sections were washed, counterstained with 4’,6-diamidino-2-phenylindole (DAPI) to visualize the nuclei, and mounted with antifade mounting medium. Fluorescence images were captured via an Olympus DP74-CU fluorescence microscope.

### Periodic acid–Schiff (PAS) staining

Distal small intestine tissue sections were stained via the periodic acid–Schiff (PAS) technique to visualize carbohydrate-rich components. Formalin-fixed, paraffin-embedded tissue sections were deparaffinized in xylene, rehydrated through a graded alcohol series, and rinsed with distilled water. The sections were then oxidized with 1% periodic acid for 10 min, followed by staining with Schiff’s reagent for 15 min. After being washed in running tap water for 10 min, the sections were counterstained with hematoxylin for 2 min to visualize the nuclei. Finally, the sections were dehydrated through a series of graded alcohols, cleared in xylene, and mounted with permanent medium. PAS-positive structures were identified by their characteristic magenta color. Images were captured via an Olympus DP74-CU microscope.

### TUNEL assay for apoptosis detection

Apoptotic cells were detected via a one-step TUNEL In Situ Apoptosis Kit (Red, Elab Fluor® 594) (catalog #E-CK-A322, Elabscience) following the manufacturer’s protocol. Briefly, formalin-fixed, paraffin-embedded distal small intestine tissue sections were deparaffinized, rehydrated through a graded alcohol series, and permeabilized with proteinase K (20 μg/mL) for 20 min at room temperature. The samples were first incubated with 100 µL of TdT equilibration buffer at 37 °C for 30 min. The equilibration buffer was then removed via absorbent paper, and 50 µL of Labeling Working Solution was added to each slide. The slides were incubated at 37 °C for 60 min in a humidified chamber protected from light. After labeling, the slides were washed three times with PBS for 5 min each, and the sections were counterstained with DAPI to visualize the nuclei. A DNase-treated section served as a positive control, whereas a negative control was prepared by omitting the TdT enzyme. The slides were mounted with antifade mounting medium, and the TUNEL-positive cells were visualized via a fluorescence microscope (Olympus, model DP74-CU).

### Tamoxifen treatment

Six-week-old Rosa26-cre^ER^ NLRC4 V341A^fl/fl^ mice were treated with tamoxifen (MedChem Express, HY-13757A, 75 mg/kg body weight) consecutively for 5 days via intraperitoneal injection to obtain global conditional NLRC4 KI mice (referred to as NLRC4 cKI), and Rosa26-cre^ER^ NLRC4^+/+^ mice treated with tamoxifen were used as wild-type controls.

### Treatment of AIFEC pups

NLRC4 KI pups received intraperitoneal (i.p.) injections of IL-18BP (ILP-H5222, Acro Biosystem, 8 mg/kg) [65], anti-TNF antibody (Infliximab, Catalog# A2019, Selleckchem, 5 mg/kg) [66], or glucose (200 mg/kg) [42] from day 1 to day 7 postbirth. Each treatment was administered once daily via a sterile syringe and needle (26–30 gauge). The injection volume was adjusted to the weight of the pups to ensure a consistent dose across all individuals. The control groups received equivalent volumes of sterile physiological saline or isotype control antibodies as indicated in the figure legends. Injections were performed in the lower right quadrant of the abdomen under aseptic conditions. Pups were monitored daily for weight, survival, and overall health. The experimental endpoints included histological analysis, cytokine measurements, and other biochemical assays, as described in the text.

### Statistics

*P* values for two-group comparisons were determined by Student’s *t* test. *P* values for body weight change and survival rate were determined by two-way ANOVA and the log-rank (Mantel‒Cox) test, respectively. All values are presented as the means ± SEMs, and *p* values < 0.05 were considered significant.

## Supplementary information

Supplemental data
