# Supplementary material for: An animal model of NLRC4-associated autoinflammation and infantile enterocolitis reveals novel therapeutic strategies
Source: Cell Mol Immunol. 2025 Oct 20;22(12):1567–80. doi: 10.1038/s41423-025-01355-x (PMC12660311; doi:10.1038/s41423-025-01355-x)
Supplement: Supplementary file 1 — Supplemental data [file 41423_2025_1355_MOESM1_ESM.docx]

**Supplementary Materials for**

**An animal model for NLRC4-associated autoinflammation and infantile enterocolitis reveals novel therapeutic strategies**

Yuhang Wang^1^, Joyce Z. Gao^1^, Prajwal Gurung^2^, Sarah P. Short^2^, Yiqin Xiong^1,3^,

Scott W Canna ^4^, Zizhen Kang^1^ ^✉^

**Affiliations**

1.Department of Pathology, University of Iowa, Iowa City, IA, USA

2.Department of Internal Medicine, University of Iowa, Iowa City, IA, USA

3.Current address: Pathology and Laboratory Medicine, Boston University, Boston, MA, USA

4.Rheumatology and Immune Dysregulation, The Children's Hospital of Philadelphia and University of Pennsylvania Perelman School of Medicine, Philadelphia, PA, USA

**Corresponding author**: Zizhen Kang, ^✉^Email: [zizhen-kang@uiowa.edu](mailto:zizhen-kang@uiowa.edu)

This PDF include:

- Supplementary Figures 1-4
- Supplementary Table 1


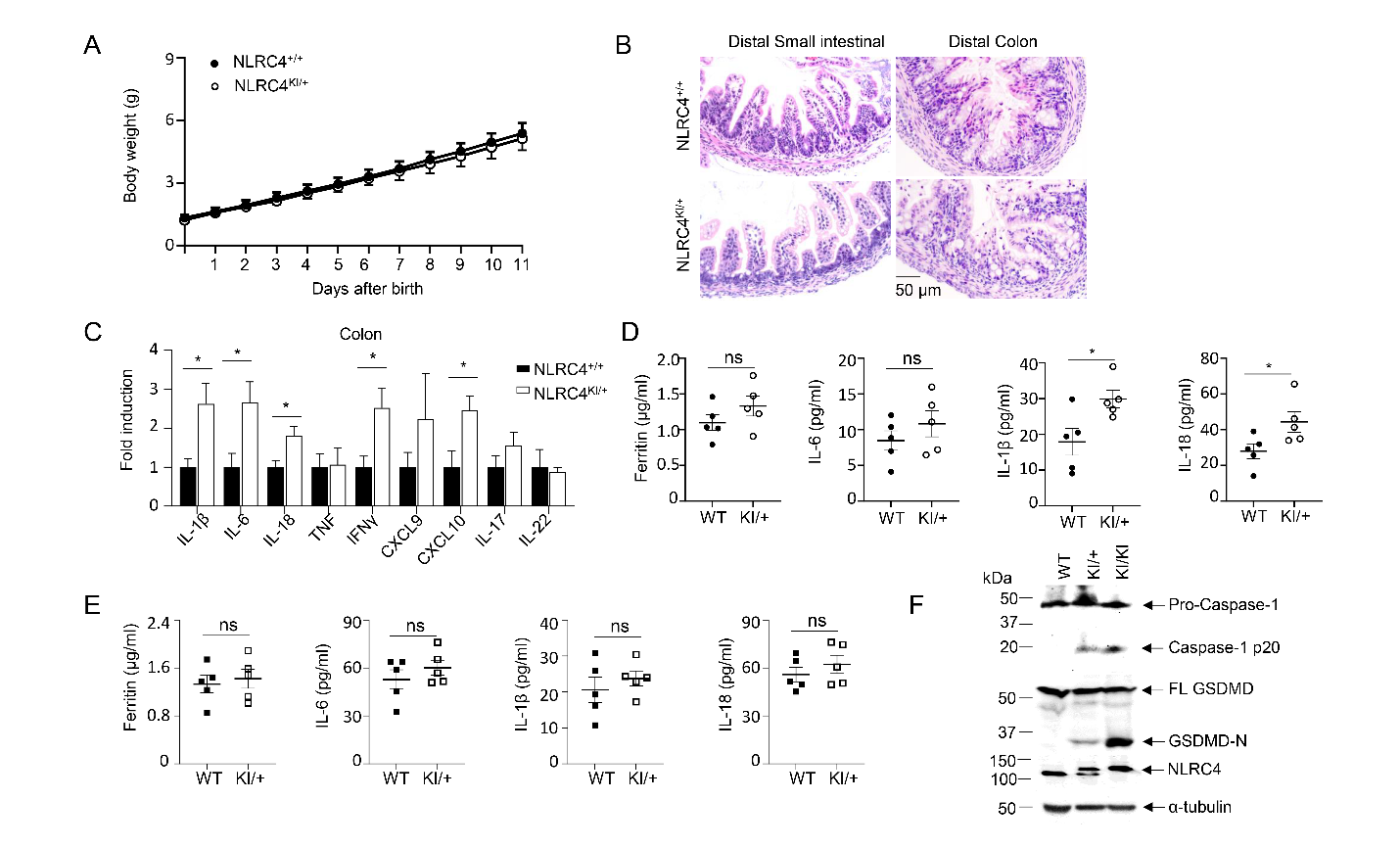


**Figure S1. NLRC4 V341A^KI/+^ mice develop mild AIFEC in infancy.** (A) Body weight of NLRC4 WT (NLRC4^+/+^) and NLRC4 KI/+ (NLRC4^KI/+^) mice after birth were plotted (n=10 per group). P values were determined by two-way ANOVA. (B) Representative H&E staining of small intestine and colon tissues from 6-day-old NLRC4 WT and KI/+ mice as indicated. (C) Colon tissues from 6-day-old NLRC4 WT and KI/+ mice were analyzed for inflammatory gene expression as indicated by real-time PCR (n=4 per group). (D) Serum ferritin, IL-6, IL-1β and IL-18 from 6-day-old NLRC4 WT and NLRC4 KI/+ mice were measured by ELISA (n=5 per group). (E) Serum ferritin, IL-6, IL-1β and IL-18 from 22-day-old NLRC4 WT and NLRC4 KI/+ mice were measured by ELISA (n=5 per group). (F) Inflammasome activation in IECs from NLRC4 WT, KI/+ and KI/KI mice was analyzed by western blot with indicated antibodies (FL GSDMD, full length Gasdermin-D; GSDMD-N, N-terminal GSDMD), α-tubulin was used as the internal control. Data are shown as mean ± SEM. p values were determined by Student’s t-test (Panels C-E), ns, p>0.05, no significant; *p<0.05. Data are representative of three independent experiments.

**
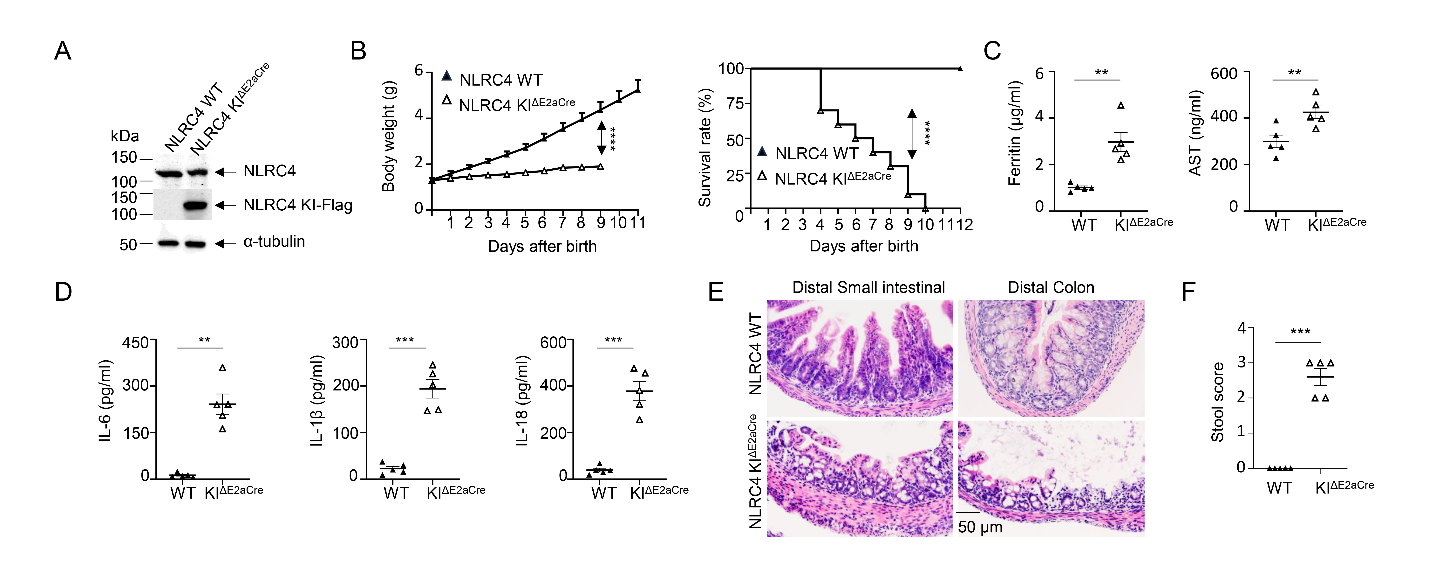
 Figure S2. Germline NLRC4 KI mice develop AIFEC independent of E2a-Cre**

E2aCre^+/-^–NLRC4 V341A^KI/+^ mice were intercrossed and generated NLRC4 V341A^KI/KI^ mice lacking E2aCre (hereafter referred to as NLRC4 KI^ΔE2aCre^) along with littermate wild-type controls (NLRC4 WT). (A) Colon tissues of NLRC4 WT and NLRC4 KI^ΔE2acre^ mice were collected at day6, western blot was performed to detect NLRC4 expression. Samples were probed by NLRC4 and Flag antibodies. α-tubulin was used as the internal control. (B) Body weight (left) and survival rate (right) of NLRC4 WT and NLRC4 KI^ΔE2acre^ mice after birth were plotted (n=10 per group). P values were determined by two-way ANOVA and Mantel-Cox test, respectively. (C) Serum ferritin and AST levels in 6-day-old NLRC4 WT and NLRC4 KI^ΔE2acre^ mice were quantified by ELISA (n=5 per group). (D) Serum IL-6, IL-1β and IL-18 levels in 6-day-old NLRC4 WT and NLRC4 KI^ΔE2acre^ mice were quantified by ELISA (n=5 per group). (E) Representative H&E staining of small intestine and colon tissues from 6-day-old NLRC4 WT and NLRC4 KI^ΔE2acre^ mice as indicated. (F) Stool scores of 6-day-old NLRC4 WT and NLRC4 KI^ΔE2a-cre^ mice (n=5 per group). Data are shown as mean ± SEM. p values were determined by Student’s t-test (Panels C, D and F), **p<0.01, ***p<0.001, ****p<0.0001. Data are representative of two independent experiments.


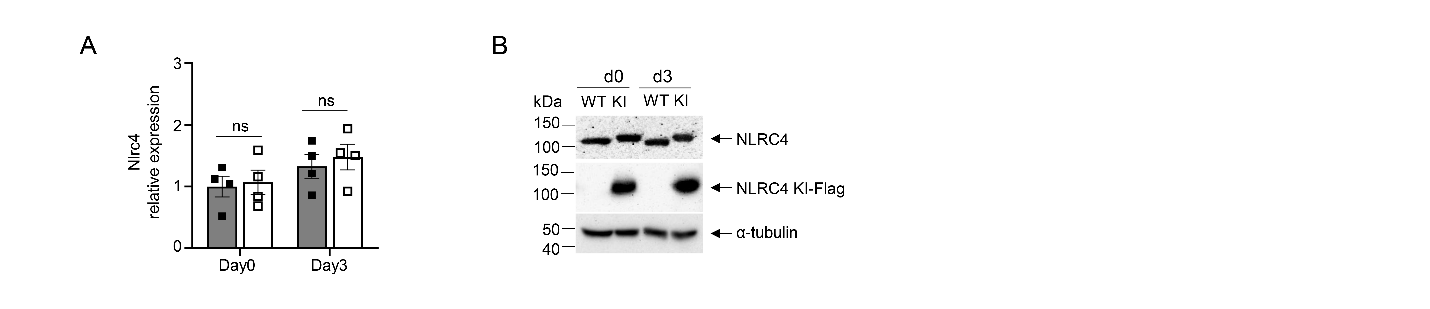
 **Figure S3. NLRC4 WT and KI mice have comparable NLRC4 expression.** Colon tissues of NLRC4 WT (NLRC4^+/+^) and NLRC4 KI (NLRC4^KI/KI^) mice were collected at day 0 and day 3, q-PCR (A) and Western Blot (B) were performed to detect NLRC4 expression. For western blot analysis, samples were probed with NLRC4 and Flag antibodies as indicated, with α-tubulin serving as the internal control. Data are representative of two independent experiments.


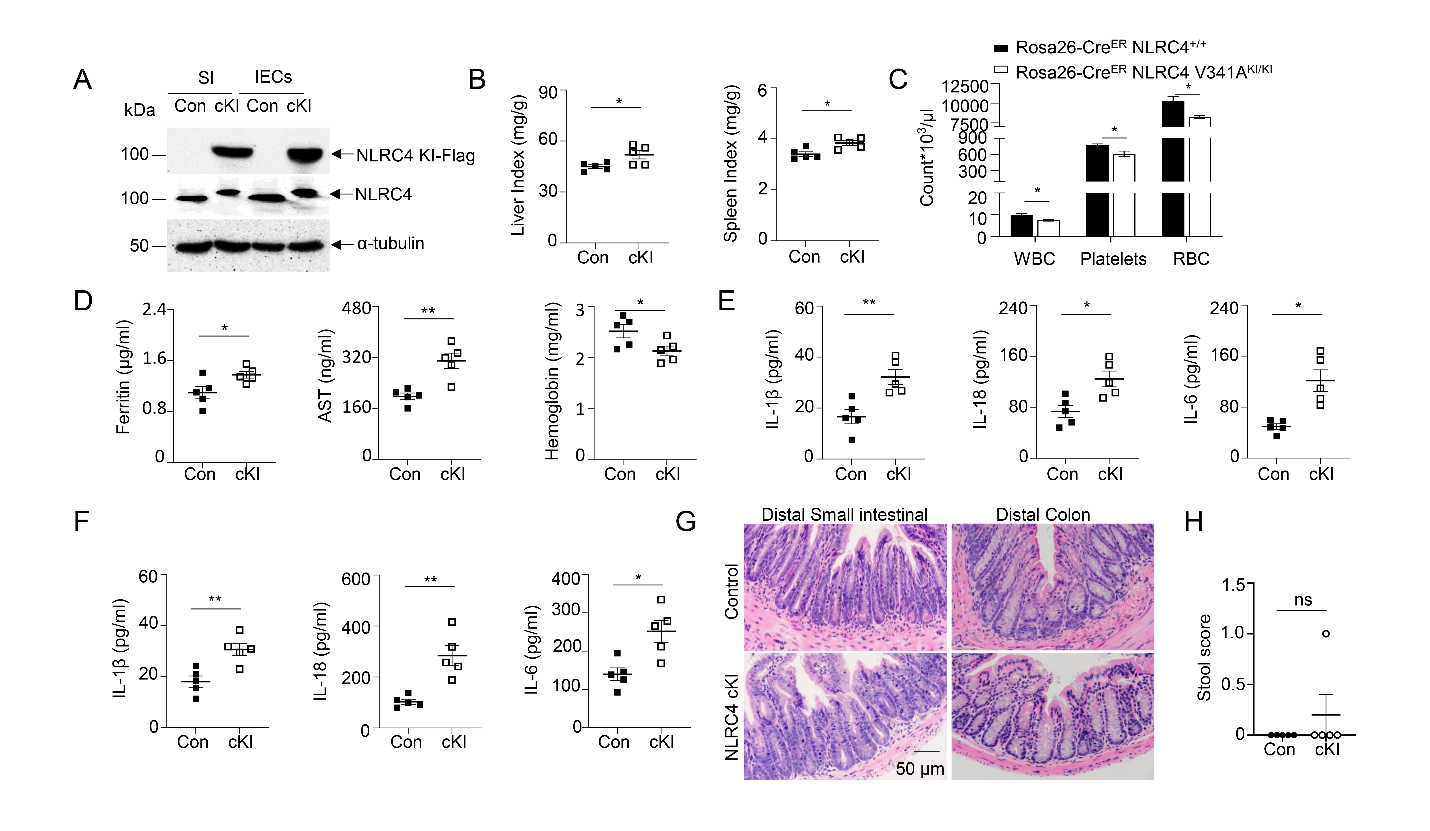


**Figure S4. Adult NLRC4 cKI mice exhibit autoinflammation with mild colitis**. 6-weeks-old Rosa26-creER NLRC4^+/+^ mice and Rosa26-creER NLRC4 V341A^fl/fl^ mice were treated with tamoxifen (75 mg/kg) consecutively for 5 days to obtain control (Con) and global conditional NLRC4 KI (cKI) mice. After treatment, mice were sacrificed at 8 weeks old for the following analysis. (A) NLRC4 cKI and control mice were genotyped by western blot. Samples from small intestines (SI), Intestinal epithelial cells (IECs) were probed by NLRC4 and Flag antibodies as indicated, with α-tubulin serving as the internal control. (B) Liver index (liver weight/body weight, Left) and spleen index (Right) of NLRC4 cKI and control mice are presented. (C) Blood cells from 8-week-old NLRC4 cKI and control mice were analyzed by flow cytometry as indicated. WBC, white blood cell; RBC, Red blood cell. (D) Serum Ferritin, AST and hemoglobin levels of NLRC4 cKI and control mice were measured by ELISA as indicated. (E) Serum IL-1β, IL-18 and IL-6 levels of NLRC4 cKI and control mice were measured by ELISA as indicated. (F) IL-1β, IL-18 and IL-6 levels in supernatants of colon explant culture from NLRC4 cKI and control mice were measured by ELISA as indicated. (G) Representative H&E staining of small intestine and colon tissues from 8-week-old NLRC4 cKI and control mice as indicated. (H) Stool scores of 8-weeks-old NLRC4 cKI and control mice. Sample size: n=5/group. Data are shown as mean ± SEM. p values were determined by Student’s t test, *p<0.05, **p<0.01. Data are representative of two independent experiments.

**Supplementary Table 1 Primer list**

|  |  | Forward primer | Reverse primer |
| --- | --- | --- | --- |
| 1 | IL-1b | ACTGTTTCTAATGCCTTCCC | TGGTTTCTTGTGACCCTGA |
| 2 | IL6 | TAGTCCTTCCTACCCCAATTTCC | TTGGTCCTTAGCCACTCCTTC |
| 3 | IL-18 | GACTCTTGCGTCAACTTCAAGG | CAGGCTGTCTTTTGTCAACGA |
| 4 | TNF | GACCCCTTTACTCTGACCCC | AGGCTCCAGTGAATTCGGAA |
| 5 | IFNɣ | TGATGGCCTGATTGTCTTTCAA | GGATATCTGGAGGAACTGGCAA |
| 6 | CXCL9 | CAGAACCTCCCACGTAGCTTTC | GCTCTGAAGATGGGATCAAGTTAATA |
| 7 | CXCL10 | CTAGTCCTAATTGCCCTTGGTCTT | AGGAGGAGTAGCAGCTGATGTGA |
| 8 | IL17 | CCTCACACGAGGCACAAGTG | CTCTCCCTGGACTCATGTTTGC |
| 9 | IL22 | GCTTGAGGTGTCCAACTTCCAG | ACTCCTCGGAACAGTTTCTCCC |
| 10 | CXCL1 | CTTGCCTTGACCCTGAAGCTC | AGCAGTCTGTCTTCTTTCTCCGT |
| 11 | IL1R | CTGTTGGTGAGGAATGTGGCTG | GGCTCAGGATAACAGGTCTGTC |
| 12 | CCL7 | CAGAAGGATCACCAGTAGTCGG | ATAGCCTCCTCGACCCACTTCT |
| 13 | COX2 | GCGACATACTCAAGCAGGAGCA | AGTGGTAACCGCTCAGGTGTTG |
| 14 | CSF1 | GCCTCCTGTTCTACAAGTGGAAG | ACTGGCAGTTCCACCTGTCTGT |
| 15 | S100A9 | TGGTGGAAGCACAGTTGGCAAC | CAGCATCATACACTCCTCAAAGC |
| 16 | NOS2 | GAGACAGGGAAGTCTGAAGCAC | CCAGCAGTAGTTGCTCCTCTTC |
| 17 | b-actin | GTGGGAATGGGTCAGAAGGA | CTTCTCCATGTCGTCCCAGT |
